# PreTIS: A Tool to Predict Non-canonical 5’ UTR Translational Initiation Sites in Human and Mouse

**DOI:** 10.1371/journal.pcbi.1005170

**Published:** 2016-10-21

**Authors:** Kerstin Reuter, Alexander Biehl, Laurena Koch, Volkhard Helms

**Affiliations:** 1 Center for Bioinformatics, Saarland University, Saarbrücken, Germany; 2 Saarbrücken Graduate School of Computer Science, Saarland University, Saarbrücken, Germany; Ottawa University, CANADA

## Abstract

Translation of mRNA sequences into proteins typically starts at an AUG triplet. In rare cases, translation may also start at alternative non–AUG codons located in the annotated 5’ UTR which leads to an increased regulatory complexity. Since ribosome profiling detects translational start sites at the nucleotide level, the properties of these start sites can then be used for the statistical evaluation of functional open reading frames. We developed a linear regression approach to predict in–frame and out–of–frame translational start sites within the 5’ UTR from mRNA sequence information together with their translation initiation confidence. Predicted start codons comprise AUG as well as near–cognate codons. The underlying datasets are based on published translational start sites for human HEK293 and mouse embryonic stem cells that were derived by the original authors from ribosome profiling data. The average prediction accuracy of true vs. false start sites for HEK293 cells was 80%. When applied to mouse mRNA sequences, the same model predicted translation initiation sites observed in mouse ES cells with an accuracy of 76%. Moreover, we illustrate the effect of *in silico* mutations in the flanking sequence context of a start site on the predicted initiation confidence. Our new webservice *PreTIS* visualizes alternative start sites and their respective ORFs and predicts their ability to initiate translation. Solely, the mRNA sequence is required as input. *PreTIS* is accessible at http://service.bioinformatik.uni-saarland.de/pretis.

## Author Summary

Ribosome profiling data and mRNA sequence features can be used to build reliable classification models with accuracies of about 80% for start codon and open reading frame prediction in human. All predicted start sites of one transcript are postulated to have the potential to initiate translation. They could, for example, be used in different tissues or in a specific cellular condition, such as stress response. Although there exist already several other approaches to predict translational initiation start sites, so far none of them considers all in– and out–of–frame AUG and near–cognate codons. The provided web service *PreTIS* considerably simplifies and assists the analysis of mRNA sequences in terms of prediction of possible translation start sites and their visualization.

This is a *PLOS Computational Biology* Methods paper.

## Introduction

Translational initiation is a more complex process than reported in common textbooks. Experimental work showed that the canonical AUG–Methionine translational start is not always used to initiate eukaryotic translation [1–5]. Further alternative codons located upstream of the annotated AUG start can also serve as additional functional start sites and form additional or alternative ORFs. Those non–AUG triplets are postulated to differ from AUG by one nucleotide and hence comprise CUG, UUG, GUG, AAG, ACG, AGG, AUA, AUC and AUU [6]. Translation can proceed in–frame as well as out–of–frame relative to the main open reading frame [7]. This can, for example, lead to (small) upstream ORFs resulting in short peptides or to extended proteins when translational initiation takes place at an in–frame start codon located upstream of the canonical start.

Ribosome profiling data provides information on the density of ribosomes located at different regions of the transcript upon application of small chemicals that block the elongation process [4, 8]. Regions which are protected by ribosomes are not digested in the next step when the mRNA is treated with nucleases [9]. These ribosome footprints (RNA) have a length of about 30 nucleotides and are sequenced after nuclease treatment and subsequently mapped to a reference genome [9]. For example, Lee *et al.* (2012) applied ribosome profiling to human embryonic kidney 293 (HEK293) cells [3]. As translational inhibitors they used Cycloheximide (CHX) and Lactimidomycin (LTM), which both bind to the ribosome E-site [3]. While CHX can bind to both, initiating and elongating ribosomes, LTM prefers initiating ribosomes with a tRNA-empty E-site [3]. Thus, by combining both inhibitors, it is possible to differentiate initiating from elongating ribosomes [3]. Lee et al. identified 16,863 potential start sites out of about 10,000 transcripts whereby start sites were allowed to be located in the 5’ UTR, at the annotated start site, in the coding region, or in the 3’ UTR, respectively.

Possible biological reasons underlying alternative translation initiation are the expansion of biological variety, regulatory processes as well as targeting of the proteins to different compartments [10–12]. Touriol *et al.* (2003) proposed that alternative translation initiation results in different proteoforms that can exhibit different functions as well as various cell localizations, which is of great importance for cell fate [12]. Some codons (e.g. AUG or CUG) are more frequently used as translation initiation starts than other codons [3, 4].

So far, several bioinformatics studies have addressed the task of predicting alternative translational start sites or ORFs. The majority of these studies only considered AUG starts. Hatzigeorgiou (2002) applied an artificial neural network (ANN) embedding a linear search for AUG starts [13]. They achieved 94% accuracy and were able to predict the correct start site in 60% of human cDNAs. Saeys *et al.* (2007) developed a meta–tool that combines three simple AUG start site predictors that consider either position–weight–matrices, k–mer frequencies or the number of stop codons downstream of a start site. This combination of several simple predictors, named *StartScan*, resulted in a sensitivity of 80%, tested on human chromosome 21 [14]. Sparks and Brendel (2008) argued that when one only searches for one translational start, predicting the leftmost (i.e. the most upstream) AUG as sole correct translational start yielded specificity and sensitivity of 94%, respectively [15]. Chen *et al.* (2014) used a flexible window and represented human DNA as k–tupels that reflect the nucleotide composition and also integrated the physicochemical properties of amino acids [16]. For AUG codons, their method achieved an accuracy of 98%. A webservice of their algorithm is available. Besides, there also exist several web–based tools for ORF identification. *ORF Finder* searches for ORFs given the accession number or sequence and the genetic code [17]. *ORF–Predictor* provides an *ab initio* prediction of ORFs based on expressed sequence tag (EST) or cDNA sequences and BLASTX alignments or intrinsic sequence signals [18].

Only few studies involved ribosome profiling data or considered in– and out–of–frame start codons differing from AUG. Ivanov *et al.* (2011) studied annotated human 5’ UTRs via sequence alignments with orthologous species followed by a manual evaluation [6] to detect non-AUG initiation in human sequences. They predicted 42 novel genes with non–AUG upstream translation initiation. For 25 of these genes non–canonical translation initiation could be experimentally validated using Western blot as well as ribosome profiling data. They also confirmed 17 alternatively translated genes that were known at this time. Crappé *et al.* (2013) applied an SVM approach to ribosome profiling data to detect small conserved open reading frames (sORFs) in mouse that code for micropeptides (10 − 100 amino acids) [19]. Baranov and colleagues (2014) used ribosome profiling data to calculate translational initiation probabilities [20]. In contrast to our work, they focused on the initiation strength of a putative start site as a function of the number of ribosome footprints.

To our best knowledge, no study so far has evaluated the general properties of human start codons considering both AUG and all near–cognate codons, in- and out–of–frame, based on start sites identified by applying ribosome profiling, and exploited this to predict the initiation confidence from the mRNA sequence.

The aim of this work was to analyze alternative translational start sites (AUG and near–cognate codons) with respect to sequence–based features to differentiate between true and false start sites. We used start sites that were identified by applying ribosome profiling to HEK293 cells [3, 5] and mouse embryonic stem cells [4] as our primary datasets. Based on mRNA sequence information we generated support vector machines as well as a linear regression model for human and mouse sequences. The learned model can then be applied to mRNA sequences not covered by ribosome profiling data or to investigate the impact of mutations in the flanking sequence context of a start site on its translation initiation confidence. Our webservice *PreTIS* visualizes putative alternative start sites and the predicted initiation confidence in human.

## Materials and Methods

### Datasets

Annotated genomic mRNA sequences for human and mouse were retrieved from Ensembl biomart (Ensembl version 77 [21]). We only included curated mRNA sequences with available mRNA RefSeq identifier (starting with NM_). It was recently shown that 85% of the start sites used to initiate translation are conserved between human and mouse [3]. Thus, we used homologous pairs of human and murine sequences to calculate the conservation of putative start codons as well as the 5’ UTR sequence conservation (see below). We identified the respective murine orthologous mRNA sequences using the approach by Ivanov *et al.* (2011) and used the first *blastn* [22] hit as the respective ortholog (default *blastn* parameters).

To identify putative start sites, each 5’ UTR was scanned for all AUGs and for alternative near–cognate start codons that differ from generic AUG by one nucleotide (CUG, UUG, GUG, AAG, ACG, AGG, AUA, AUC und AUU) and that are located either in–frame or out–of–frame with the main open reading frame. Different sequence–based features were then calculated for all putative start codons that have a downstream in–frame stop codon.

To establish reliable true positive and true negative translational start site datasets for training and testing purposes, we used the findings from different ribosome profiling experiments [3–5]. Each dataset was analyzed independently. Note that the datasets used here contain translational start sites derived from ribosome profiling data by the original authors (gene accession number, position relative to annotated start site, codon). We did not include raw ribosome profiling (footprint) data in our approach. In total, we trained two start site prediction models: a human prediction model based on the HEK293 dataset [3] and a mouse prediction model based on the Mouse ES dataset [4]. The third HEK293–AUG dataset [5] was used as validation set to further evaluate the reliability and robustness of the developed prediction model.

**Fig 1:**
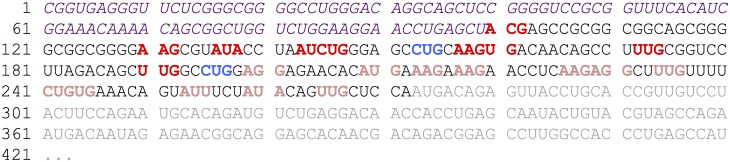
Example mRNA sequence showing the categorization of true positive and true negative start sites. Suppose that a ribosome profiling experiment detected the following start sites for a given mRNA sequence: CUG at position -78 and CUG at position -120 (blue colored codons). These start sites were then assumed to be true positive start sites. In consequence, all near-cognate start sites not listed in the ribosome profiling dataset and upstream of the most downstream reported true start site were assumed to be true negatives (dark red colored codons). The light red colored codons are start sites not considered as false starts in the analyses since they are located downstream of the most downstream reported true start site. Note that the grey colored downstream part depicts the annotated CDS sequence whereas the italic (purple) upstream part marks the -99 upstream window needed to calculate some of the features (see below). All marked start sites (true positive and true negative) exhibit a surrounding window of ±99 nucleotides as well as a downstream in–frame stop codon. In total, this mRNA sequence would provide 2 true start sites and 9 false start sites out of 23 putative starts.

For training and testing of every classifier, we considered each start site (AUG and near-cognate) that matched a start codon found by ribosome profiling as a true start. False start sites were defined as follows: remaining candidate start sites (AUG and near-cognate) that were not detected by ribosome profiling and that are, based on the assumption of a linear scanning model, located at least 99 nts downstream of the transcription start site as well as upstream of the most downstream reported true translation initiation start. Fig 1 shows an example mRNA sequence that illustrates the grouping of true positive and true negative start sites for training and testing purposes based on ribosome profiling data. This start sites categorization was executed for each of the three datasets, each time based on the individual ribosome profiling experimental results [3–5].

### Features based on mRNA sequence information

All features used here are solely based on information derived from the mRNA sequences.

#### Position–weight–matrix (PWM)

In mammalian cells, some codons (e.g. AUG and CUG) are more frequently used to initiate translation compared to other codons (e.g. AUA or AGG) [3, 4]. In the HEK293 dataset used here, 26.1% of the reported upstream initiation start codons are AUGs and 29.8% are CUGs [3], see S1 Fig. The start codon information was considered using position-weight-matrices (PWMs). To account for the important role of the flanking sequence context for translation initiation, we considered a window ranging from -15 to +10 with respect to the start site in a set of sequences *S*. First we calculated from the data in the training set a position–frequency–matrix (PFM) with the nucleotides *nt* ∈ {A,C,U,G} as the rows and the sequence position *i* as the columns. The matrix entries were filled by dividing the sum of occurrences of a nucleotide at position *i* by the total number of sequences contained in *S*. The PWM was then calculated by dividing each entry in the PFM by the respective nucleotide background frequency and taking the natural logarithm, i.e.

$${\text{PWM}}_{\left(nt,i\right)}=log\left(\frac{PFM_{\left(nt,i\right)}}{bg_{nt}}\right)$$

where the background frequency *bg*_*nt*_ is defined as the *actual* nucleotide frequency of the 5’ UTR in *S*. We calculated three PWMs, one based on the true start sites (*PWM*_*positive*_), one based on the false start sites (*PWM*_*negative*_), and one based on the log–ratio between true and false start sites (*PWM*_*ratio*_) in the training set. The *PWM*_*score*_ for a sequence *s* was then computed as

$$PWM_{score}\left(s\right)=\sum_{i=0}^{len(s)}PWM_{nt_{i},i}$$

where *nt*_*i*_ is the nucleotide occurring at position *i* in sequence *s*. With *PWM*_*positive*_, a *PWM*_*score*_ greater than zero indicates that the given sequence *s* is more likely a true start than a false start while a *PWM*_*score*_ less than zero suggests a higher probability of being a false start site.

#### Start site conservation

To calculate the conservation of a putative start site, sequence alignments between pairs of human and mouse sequences (5’ UTR and CDS), found by applying *blastn*, were generated using MUSCLE [23]. For this, 5’ UTR and CDS were translated into all three possible reading frames and were aligned accordingly. We then translated the protein alignment back into the associated (gap-free) nucleotide alignment (compare with [3]). A human start site was assumed to be conserved if it shares the same codon or amino acid with the murine ortholog at the respective position. This yielded two binary features: codon and amino acid conservation. We also calculated the average degree of 5’ UTR sequence conservation, using the human–mouse mRNA sequence alignment. For this we divided the number of matching nucleotides by the length of the 5’ UTR sequence. Gaps were ignored.

#### Start codon flanking sequence context

The flanking sequence context was assessed in two ways where we considered either only the positions -3R (R = purine) and +4G, which were determined to be crucial for initiation [24, 25], or experimentally determined translational start codon efficiencies [26]. In the first approach, the Kozak sequence context was discretized into strong (A or G at -3 and G at +4), intermediate (A or G at -3 and no G at +4), weak (no A and no G at -3 and G at +4) and no Kozak context. These categories were presented as the values 1 (no), 2 (weak), 3 (intermediate) and 4 (strong). In the second approach, we used the raw translation efficiency values reported by Noderer *et al.* (2014) as feature for the respective flanking sequence context of a start site. These authors investigated the translational efficiency of all possible 11–nt–long (position -6 to +5) flanking sequence contexts around the AUG translational start using high–throughput sequencing combined with fluorescence signaling [26]. We assumed that alternative starts behave similarly as AUG codons and therefore use the same translational efficiency values for the alternative starts.

#### Minimum free energy of mRNA secondary structure

Secondary structure is an important factor for translation initiation [25, 27, 28]. Dependent on the propensity of the mRNA secondary structure downstream of a putative start codon, the ribosome scanning in downstream direction can pause and translation is initiated [28]. It was shown that a secondary structure with a minimum free energy of $\Delta G=-19\frac{kcal}{mol}$ that starts 12–15 nt downstream of the translational start site can prevent leaky scanning and compensate for an unfavorable flanking sequence context [28, 29]. A secondary structure starting 14 nts from the translational initiation site was observed to have the largest effect [28]. Here, we considered different windows for calculating the minimum free energy of the secondary structure and then selected the most suitable one to differentiate between true and false start sites: a 60–nt window starting at position +14, a 60–nt window starting at position +20, a window from position -10 to +50 and a window from -50 to +50. Minimum free energies were calculated using RNAfold [30].

#### GC–content

It was shown that the GC–content continuously decreases from 5’ UTR across the CDS to the 3’ end in human [31]. We therefore analyzed whether the GC–content differs between true and false start sites using the same windows as for the minimum free energy (see above). Note that the minimum free energy of an mRNA secondary structure and its GC–content are related to each other since G–C pairs possess a higher degree of stability than A–U pairs due to their additional hydrogen bond [32].

#### Open–reading–frame (ORF) length

It appears plausible that the length of ORFs that code for functional proteins is generally longer than the ones resulting from arbitrary start sites in the mRNA sequence. Therefore, we also considered the length of the putative open reading frame.

#### 5’ UTR nucleotide distribution

As mentioned before, the GC–content varies between 5’ UTR and CDS. If a part of the annotated 5’ UTR is actually used as CDS this may result in a different nucleotide composition compared to the actual 5’ UTR. Therefore, we calculated the percentage of all four nucleotides (e.g. $\frac{\#A}{5^{\prime }UTR\;length}$) in the entire 5’ UTR. This resulted in four additional features.

#### 5’ UTR length

We also tested the 5’ UTR length with respect to significant differences between true and false start sites by defining the 5’ UTR (nucleotide) length as further feature.

#### K–mer search

We counted the frequency of all possible k–mers of length *k* = 1 (position–specific k–mers) and *k* = 3 (codon and respective amino acid k–mers) in a window from -99 to +99 around the start site. k-mers were defined as all possible combinations of subsequences of length k, given an alphabet, here nucleotides {*A*, *C*, *U*, *G*}. We considered in–frame and out–of–frame k–mers as well as k–mers upstream and/or downstream of the start site as suggested in [33]. In total, this yielded 1,229 k–mers: position–specific k–mers in the predefined window of ±99 amount to 198 positions × 4 nucleotides = 792 (e.g. “K-mer: position -12 is C”), 64 codons × 5 (counted in the complete ±99 region, the upstream region, the downstream region as well as in–frame–downstream and in–frame–upstream) = 320, 20 amino acids × 5 = 100, 1 stop codon × 5 = 5, and 4 nucleotides (*k* = 1) × 3 (complete ±99 region, upstream region and downstream region) = 12. This sums up to 792 + 320 + 100 + 5 + 12 = 1,229 k–mers.

In total, we considered 1,252 features, with three features based on PWMs, 20 biologically–motivated features (e.g. conservation or the flanking sequence context) and 1,229 features found by a k–mer search for *k* = 1 and *k* = 3.

### Regression approach

**Fig 2:**
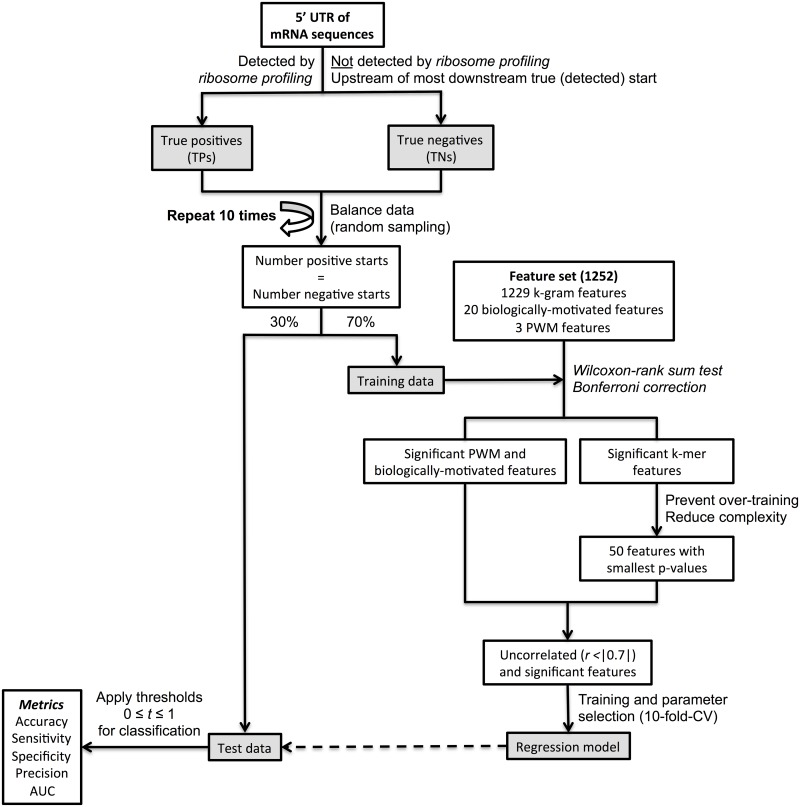
Flowchart of the regression approach. Data balancing was repeated ten times to investigate model robustness. Significant features were identified by the Wilcoxon-rank sum test.

The prediction approach, shown in Fig 2, was applied to the human HEK293 [3] and mouse ES datasets [4].

First, as mentioned, all putative start sites in the 5’ UTR were defined as true positives or true negatives based on the reported ribosome profiling data and their location in the mRNA sequence. We then balanced the size of the dataset so that it contains the same number of true and false start sites by randomly under–sampling from the larger dataset. We repeated the data balancing as well as the assignment of random training and test sets 10 times to evaluate the model robustness and reported the average performance. We applied Wilcoxon–rank sum test and Bonferroni correction (with a significance threshold of $\frac{0.01}{1,252}=8\times 10^{-6}$, with the total number of features as the denominator) to test for the statistical significance of the biological, the k–mer, and the PWM features to differentiate between true and false start sites. We subsequently calculated all pairwise Pearson correlations between the significant biological and PWM features as well as for the 50 most significant k–mer features and only used uncorrelated (|*r*| < 0.7) features in the training step. If two or more features were correlated, the one with the smallest p–value was used. The PWMs were calculated in each training step iteration to guarantee that the test set is independent on the calculated PWMs. All features were normalized (mean zero and unit variance) to ensure comparability.

Next, we generated simple linear as well as support vector (SVR) regression models on 70% of this data and tested them on the remaining 30% of the data, using three different kernels for the SVM approach: linear, radial basis function (RBF) and polynomial. We applied 10–fold cross–validation to find the best penalty parameter *C* in [0.1, 1, 10, 100] and *ϵ*-tube parameter in [0.01, 0.1, 1, 10] for the training data set when applying SVR models. The remainder of parameters were kept at default values.

Since we applied a regression approach, we subsequently applied 100 classification thresholds 0.0 ≤ *t* ≤ 1.0 in steps of 0.01 to the predicted output *out*_*pred*_ in order to classify every start site as true or false based on its model outcome and the given threshold. These thresholds can be interpreted as initiation confidence where start sites with a regression value *out*_*pred*_ ≥ *t* are predicted as true start sites and the ones with *out*_*pred*_ < *t* as false start sites. If a start site is predicted with an initiation confidence > 1, we substituted this value by 1. The same holds for start sites with a predicted confidence < 0, which were substituted with zero. We then compared the predicted class with the correct class and used the common measures accuracy, specificity, sensitivity, precision and area under the curve (AUC) as metrics for model assessment. The final model for the prediction of new mRNA sequences and for a SNP analysis was subsequently determined by comparing different model performances.

The implementation was done in Python (version 2.7) and using the Scikit–learn package (version 0.17) for the machine learning part [34].

### *In silico* SNP analysis

To investigate the effect of putative single nucleotide polymorphisms (SNPs) within the flanking sequence context of the start sites (position -15 to +10), we substituted (*in silico*) one nucleotide position at a time by all 3 remaining nucleotides, yielding 75 different contexts (the start codon itself was not mutated). We then recalculated the needed sequence features to investigate the mutational impact and subsequently applied our final prediction model to all contexts. We then report the effect of these substitutions on the predicted initiation probabilities.

## Results

In this work we used ribosome profiling data from HEK293 cells [3] and mouse ES cells [4] to analyze sequence encoded differences between true and false translational initiation sites located in the mRNA 5’ UTR. A third dataset, only containing AUG starts, was used as validation set [5]. Calculated sequence-based features were subsequently used to build a prediction model. In the following, we present the datasets used, the results of the regression approach, its application and an implementation as webservice *PreTIS*.

### Filtered dataset

The start sites reported by ref. [3–5] based on ribosome profiling data were filtered to include only starts matching AUG and near–cognate codons in the 5’ UTR. For HEK293 cells [3], this yielded 4,482 true start sites (i.e. reported in the experimental analysis) and 49,520 false start sites in 3,566 mRNAs. For mouse ES cells [4], this gave 3,009 true start sites and 19,864 false start sites in 1,632 mRNAs. True (reported) starts were assumed to be true positives (TP) and false (not reported and upstream of the most downstream reported) starts were assumed to be true negatives (TN). For comparison, we also included a smaller dataset of Ohler and colleagues [5] who only determined AUG starts in HEK293 cells. Table 1 displays the three datasets. All reported analyses are based on these filtered datasets.

**Table 1:** Datasets used in this study.

Cell line	Description	Genes	Start codons	TPs	TNs	Used for	Source
HEK293	Human embryonic kidney cells	3,566	AUG and near-cognate	4,482	49,520	Human prediction model	[3]
HEK293	Human embryonic kidney cells	391	AUG	332	447	Validation set	[5]
Mouse ES	Mouse embryonic stem cells	1,632	AUG and near-cognate	3,009	19,864	Mouse prediction model	[4]

Three different datasets were used in this study to establish a human and mouse prediction model and to cross-validate the regression models. The numbers indicate the filtered start sites used in the prediction approach.

Among the considered AUG and near-cognate start codons, AUG (human: 26%, mouse: 16%), CUG (human: 30%, mouse: 34%) and GUG (human: 13%, mouse: 19%) were the most prevalent translational start codons, see S1 Fig. Thus, CUG and GUG are more often used in mouse compared to human. This is in accordance with [3, 4] and shows that the start codon itself is very important for translational initiation.

### Regression approach

Table 1 illustrates that the negative sets outnumber the positive sets by factors of 7 (mouse ES) and 11 (HEK293). To avoid a class size dependent bias, we randomly under–sampled the same number as true positive start sites from the true negative set. Next, we trained on 70% and tested on 30% of the data (randomly assigned).

Table 2 lists the performance of human and mouse models together with the optimal thresholds *t*. All human models perform very similarly with accuracies of about 80% while the average performance of the mouse model is lower with average accuracies of about 76%, see Table 2. We also computed receiver operating characteristic (ROC) curves and the associated area under the curve (AUC). In accordance with the other metrics, also the AUC values were satisfactory with average values of about 80% and 76% for the human and mouse models, respectively (Table 2).

**Table 2:** Evaluation of the regression approach.

	Accuracy	Specificity	Sensitivity	Precision	AUC	Threshold
**HEK293**						
Linear SVR	0.80±0.01	0.80±0.01	0.81±0.01	0.80±0.01	0.80±0.01	0.62±0.01
RBF SVR	0.82±0.01	0.81±0.01	0.83±0.02	0.82±0.01	0.82±0.01	0.55±0.02
Polynomial SVR	0.80±0.01	0.80±0.01	0.81±0.02	0.80±0.01	0.80±0.01	0.59±0.02
Linear Regression	0.80±0.01	0.80±0.01	0.81±0.01	0.80±0.01	0.80±0.01	0.55±0.01
**Mouse ES**						
Linear SVR	0.75±0.01	0.75±0.01	0.76±0.01	0.75±0.01	0.76±0.01	0.65±0.03
RBF SVR	0.76±0.01	0.76±0.01	0.76±0.02	0.76±0.01	0.76±0.01	0.58±0.03
Polynomial SVR	0.75±0.02	0.75±0.01	0.76±0.02	0.75±0.02	0.75±0.02	0.62±0.03
Linear Regression	0.76±0.01	0.75±0.01	0.76±0.01	0.75±0.01	0.76±0.01	0.55±0.01

The prediction was repeated 10 times to evaluate the model robustness. Shown are the average performance measures.

#### Best performing prediction model

Since all models gave a very similar performance with accuracies of about 80%, we decided to choose the simple linear regression model that can be interpreted well. The best performing and nicely balanced human linear regression model was obtained in run 2 (the prediction was repeated 10 times). This model had an accuracy of 83%, a sensitivity of 84%, a specificity of 82% and a precision of 83% on the test data. It was then applied to predict unknown start sites of a gene of interest and to conduct an *in silico* mutation analysis. Moreover, it is embedded in the webservice *PreTIS*. Therefore, this model is analyzed in more detail in the following.

**Fig 3:**
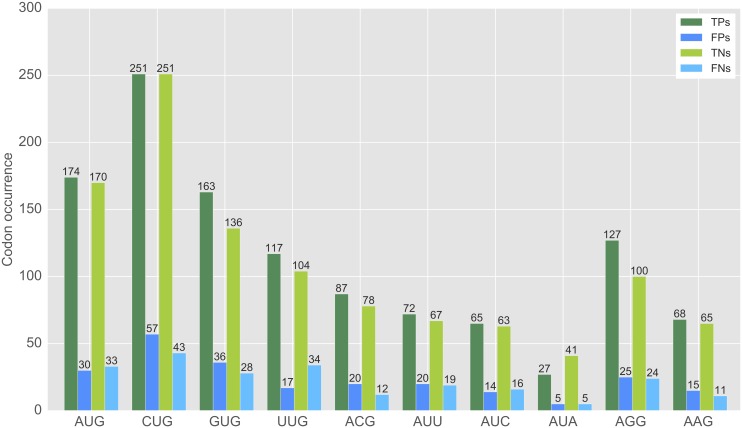
Codon distribution of the test samples in the best performing human model. AUG, CUG and GUG were the most prevalent true positive start sites (*t* = 0.54).

Fig 3 displays the predicted codon distribution when applying the best performing linear regression model of run 2 to the mRNA sequences in the test set and using the threshold *t* = 0.54 that gave the best overall performance.

The distribution of predicted codons agreed with the preferences found experimentally [3, 4]: AUG and CUG are the most prevalent start codons whereas AUA or AAG are more often classified as true negatives. Nevertheless, our predictor also detects true negative AUGs and CUGs and true positive AUAs and AAGs.

The features that were used to build this prediction model are displayed in Table 3. The most significant feature is the length of the 5’ UTR (*p* < 10^−310^). The 5’ UTR was found to be shorter on average for true starts sites (414±270 nts) compared to false start sites (675±545 nts). The second most significant biologically–motivated feature with a p–value of *p* = 8.2 × 10^−190^ was the conservation of the 5’ UTR. The values of 0.4±0.16 for the true start sites and 0.33±0.16 for false start sites suggest that 5’ UTRs harboring true start sites are in general more conserved. Another highly significant feature (*p* = 5.1 × 10^−144^) was the number of upstream AUGs. Considered false start sites had more upstream AUGs (0.59±0.9) than considered true start sites (0.22±0.57), see Table 3. This can be explained as follows: if AUG is located upstream of another putative start site, the linear scanning model of Kozak [35] implies that it is more probable that the AUG is used as start site instead.

**Table 3:** Mean value and standard deviation of the 44 features that were used in the best human model.

	Feature	True starts	False starts	P–value
1.	**5’ UTR length**	414.41±270.48	675.41±545.35	< 10^−310^
2.	**5’ UTR conservation**	0.4±0.16	0.33±0.16	8.2 × 10^−190^
3.	**PWM positive**	2.75±1.5	-0.14±2.82	5.5 × 10^−173^
4.	K-mer: upstream AUG	0.22±0.57	0.59±0.9	5.1 × 10^−144^
5.	**5’ UTR: percentage A**	0.18±0.05	0.2±0.05	9.6 × 10^−100^
6.	**Kozak sequence context**	2.67±1.07	2.3±1.11	9.2 × 10^−95^
7.	**Translational efficiency of flanking sequence**	83.75±20.11	77.12±21.4	1.1 × 10^−83^
8.	K-mer: position -12 is C	0.13±0.34	0.3±0.46	2.7 × 10^−77^
9.	K-mer: upstream Asparagine	1.25±1.37	1.61±1.61	4.0 × 10^−43^
10.	K-mer: downstream AUG	1.14±1.15	0.92±1.1	9.2 × 10^−41^
11.	K-mer: upstream A	17.24±7.43	18.81±7.89	4.0 × 10^−40^
12.	K-mer: in-frame upstream Alanine	3.69±2.6	3.16±2.29	4.0 × 10^−37^
13.	K-mer: upstream Alanine	10.27±4.5	9.38±4.6	6.2 × 10^−37^
14.	**5’ UTR: percentage G**	0.32±0.06	0.31±0.05	7.1 × 10^−37^
15.	**Codon conservation**	0.23±0.42	0.12±0.32	3.2 × 10^−36^
16.	K-mer: position -3 is A	0.31±0.46	0.2±0.4	3.4 × 10^−35^
17.	K-mer: upstream CCG	2.98±2.43	2.56±2.31	7.1 × 10^−34^
18.	K-mer: downstream CCA	2.04±1.54	1.75±1.45	1.1 × 10^−32^
19.	K-mer: position -12 is A	0.3±0.46	0.19±0.4	4.0 × 10^−32^
20.	K-mer: in-frame upstream Methionine	0.07±0.29	0.2±0.48	3.3 × 10^−31^
21.	K-mer: upstream Arginine	12.15±4.34	11.33±4.64	1.5 × 10^−29^
22.	K-mer: upstream Histidine	1.7±1.52	1.97±1.65	2.2 × 10^−27^
23.	K-mer: GCC	6.4±3.87	5.77±3.75	1.1 × 10^−25^
24.	K-mer: position 4 is G	0.37±0.48	0.28±0.45	2.3 × 10^−25^
25.	K-mer: upstream Threonine	3.56±2.08	3.91±2.19	4.9 × 10^−25^
26.	K-mer: upstream CGG	3.14±2.51	2.77±2.41	3.2 × 10^−24^
27.	K-mer: upstream C	30.4±8.98	28.96±9.04	1.0 × 10^−23^
28.	K-mer: position -2 is G	0.23±0.42	0.32±0.47	1.2 × 10^−23^
29.	K-mer: upstream Stop	2.3±1.71	2.66±2.0	1.4 × 10^−23^
30.	K-mer: UAG	1.34±1.2	1.57±1.35	5.6 × 10^−23^
31.	K-mer: upstream CAU	0.58±0.85	0.73±0.95	3.4 × 10^−22^
32.	K-mer: upstream Serine	9.44±3.29	8.93±3.14	5.7 × 10^−22^
33.	K-mer: downstream Glutamine	3.57±2.01	3.26±1.88	2.4 × 10^−21^
34.	K-mer: AGG	4.29±2.51	4.7±2.69	2.1 × 10^−20^
35.	K-mer: AGC	4.4±2.43	4.02±2.19	2.1 × 10^−20^
36.	K-mer: downstream ACC	1.45±1.26	1.27±1.17	2.0 × 10^−19^
37.	K-mer: UAA	1.22±1.42	1.51±1.76	6.2 × 10^−19^
38.	K-mer: downstream Proline	9.3±5.63	8.56±5.47	3.5 × 10^−18^
39.	K-mer: upstream CAA	0.75±0.92	0.91±1.06	1.3 × 10^−17^
40.	K-mer: in-frame upstream Histidine	0.54±0.77	0.67±0.87	1.7 × 10^−17^
41.	K-mer: upstream GAU	0.63±0.85	0.77±0.96	2.1 × 10^−16^
42.	K-mer: in-frame upstream GCC	1.21±1.4	1.02±1.22	6.7 × 10^−16^
43.	K-mer: in-frame upstream GCG	1.14±1.42	0.97±1.27	6.2 × 10^−14^
44.	**PWM negative**	1.94±1.34	1.59±1.09	1.6 × 10^−08^

**Fig 4:**
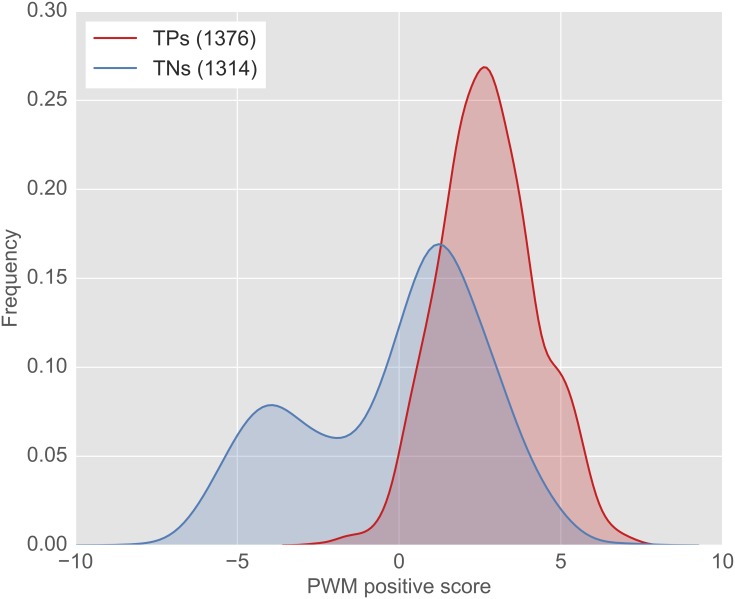
Frequency distribution of *PWM*_*positive*_ scores for the test samples of the best performing run 2. The PWM was established using the true start sites in the training data of run 2. The difference between TPs and TNs was found to be highly significant (*p* = 5.5 × 10^−173^, Wilcoxon–rank sum test).

Mean value and standard deviation of the 44 features that were used in the best human model (biologically-motivated and PWM features are shown in bold). All 4,482 true and 49,520 false start sites were considered for this analysis. All listed features showed significant differences between true and false start sites (P–values < 1.6 × 10^−8^). Note that due to numerical reasons, very small p–values (< 10^−310^) are represented as 0.0 in python programming language (*scipy version 0.17.0*). The PWM–scores are based on the test data (compare to Fig 4).

*PWM*_*positive*_ was also found to be highly significant (*p* = 5.5 × 10^−173^). The PWMs were recalculated for each training sample to achieve unbiased test samples in each run. The background frequency of the best performing run 2 is *bg*_*A*_:0.16, *bg*_*C*_:0.29, *bg*_*U*_:0.21 and *bg*_*G*_:0.34, while the average background frequency of all training and test runs was calculated as *bg*_*A*_:0.21±0.06, *bg*_*C*_:0.27±0.06, *bg*_*U*_:0.22±0.06 and *bg*_*G*_:0.3±0.06. Thus, as expected [31], Guanine and Cytosine are prevalent in the 5’ UTR. Fig 4 shows the PWM scores calculated for the test samples in the run with best overall performance (run 2) based on the PWM generated using the true training samples (*PWM*_*positive*_) in this run. The scores of the true (test) start sites were significantly higher (2.75 ± 1.5) than those of the false (test) start sites (−0.14 ± 2.82).

Interestingly, the distribution of the false start sites was found to be bimodal. Thus, one might speculate that some of these considered false start sites with higher PWM values (i.e. start sites not found by the ribosome profiling technique and located upstream of the most downstream reported true start, which are therefore considered as true negative starts, see also Fig 1) might be used as actual start sites in different cell types or cellular conditions. This also explains the overlap between the true positive and true negative start sites in Fig 4.

Another biologically important feature that also represents the flanking sequence context is the “Kozak sequence context” feature (see methods) with a p–value of 9.2 × 10^−95^. As expected from experimental findings [35], true start codons more often exhibit a strong or intermediate Kozak context compared to false start sites that often show no Kozak context at all, see Table 3 and S2 Fig. This is also in agreement with the observation that A at position -3 (*p* = 3.4 × 10^−35^) and G at position +4 (*p* = 2.3 × 10^−25^) were found (by the k–mer search) to be important for translational initiation. Similarly, the translational efficiency of the flanking sequence context, experimentally investigated in [26], was also highly significant (*p* = 1.1 × 10^−83^). The average efficiency of true start sites is, as expected, higher than the one calculated for the false start sites. Moreover, true start codons were found to be more often conserved between human and mouse sequences compared to false start sites (*p* = 3.2 × 10^−36^). Start site conservation was also mentioned in the original publication of the HEK293 dataset we used here [3].

Many significant features detected by the k–mer search contained upstream G–C–patterns (e.g. “K-mer: upstream CCG” or “K-mer: upstream CGG”) at higher frequencies for true start sites compared to false start sites. This reflects the generally higher GC–content in the 5’ UTR compared to the CDS and is in accordance with the finding that the GC–content decreases from the 5’ UTR to the CDS [31].

Consistent with the p–values of the features used in the best performing human linear regression model are the feature (weight) coefficients determined by the model training step, see S3 Fig. The highest coefficients were assigned to the *PWM*_*positive*_ and the number of upstream AUGs (“K-mer: upstream AUG”).

### Transferability of the prediction model

To investigate the transferability of our best human prediction model, we analyzed its performance using the mouse ES data as well as the HEK293–AUG dataset, see Table 4. With the threshold of *t* = 0.54 that was found to be optimal for the trained HEK293 dataset, we obtained for the mouse ES dataset an accuracy of 76%, a sensitivity of 72% and a specificity of 77%. By scanning all possible thresholds, see S4(A) Fig, we found that *t* = 0.52 yields a more balanced performance of 75%, 76% and 74% for accuracy, sensitivity and specificity, respectively. Decreasing the threshold seems to be advantageous for the mouse data set, since some true positives seem to possess weaker features for translational initiation (e.g. a weak flanking sequence context or a less common initiation codon), but are nevertheless true positive starts.

**Table 4:** Performance of the best human HEK293 model applied to the mouse ES and human HEK293–AUG datasets.

**Unbalanced datasets**								
	**Mouse ES**		**Mouse ES**		**HEK293–AUG**		**HEK293–AUG**	
Threshold	*t* = 0.54		*t* = 0.52		*t* = 0.54		*t* = 0.65	
	TP	TN	TP	TN	TP	TN	TP	TN
Predicted positive	2,161	4,569	2,273	5,072	257	249	207	160
Predicted negative	848	15,295	736	14,792	75	198	125	287
Total	3,009	19,864	3,009	19,864	332	447	332	447
Accuracy	0.76		0.75		0.58		0.63	
Sensitivity	0.72		0.76		0.77		0.62	
Specificity	0.77		0.74		0.44		0.64	
Precision	0.32		0.31		0.51		0.56	
**Balanced datasets**								
	**Mouse ES**		**Mouse ES**		**HEK293–AUG**		**HEK293–AUG**	
Threshold	*t* = 0.54		*t* = 0.52		*t* = 0.54		*t* = 0.64	
	TP	TN	TP	TN	TP	TN	TP	TN
Predicted positive	2,161	689	2,273	763	257	185	211	125
Predicted negative	848	2,320	736	2,246	75	147	121	207
Total	3,009	3,009	3,009	3,009	332	332	332	332
Accuracy	0.74		0.75		0.61		0.63	
Sensitivity	0.72		0.76		0.77		0.64	
Specificity	0.77		0.75		0.44		0.62	
Precision	0.76		0.75		0.58		0.63	

We then applied our best regression model to the start sites reported in the HEK293–AUG dataset that only contains AUG starts [5]. The categorization of true positive and true negative start sites was conducted as above for the HEK293 dataset (see Fig 1), with the only difference that the HEK293–AUG dataset only contains AUG start sites instead of AUG and all near-cognate codons. Thus, we defined again the false start sites as all AUG starts located in the 5’ UTR that were not detected by ribosome profiling and are located upstream of the most downstream true start site.

Differentiating only between true and false AUG start sites is particularly difficult because the AUG itself is a very strong signal for a true start site and just by random chance there might be AUGs with, for example, good flanking sequence, which are not used as translational start sites (or are not reported in the dataset). Moreover, our prediction model was trained on all possible cognate codons instead of AUG alone.

Our best model with the determined threshold of *t* = 0.54 detected 77% of the true AUG starts in the HEK293–AUG dataset (sensitivity of 77%). Nevertheless, the specificity of this prediction is only 44% and thus the overall accuracy is only slightly better than a random decision (58%), compare to Table 4. However, when increasing the threshold from *t* = 0.54 to *t* = 0.65, we were able to increase the overall accuracy to 63%. A threshold of *t* = 0.65 was found to be optimal for this dataset, see S5(A) Fig.

Problematic was here the precision (i.e. the number of true positives out of all samples classified as positive ($\frac{TPs}{TPs+FPs}$)). Many starts that we assumed to be true negatives actually show properties of true positives and are therefore classified as false positives. Especially if the dataset is highly unbalanced (e.g. the number of mouse ES true starts is only 15% of the false start sites) this effect has a strong influence on the precision. When we balanced our datasets, the precision increased drastically from 31% to 75% for the mouse ES dataset and *t* = 0.52 and from 56% to 63% for the HEK293–AUG dataset and *t* = 0.64, see Table 4.

### Applications of the prediction model

The established prediction model can, for example, be used to predict translational start sites which are not covered by ribosome profiling experiments or to analyze the impact of mutations in the flanking sequence around the start site.

#### Prediction of unknown start sites

**Fig 5:**
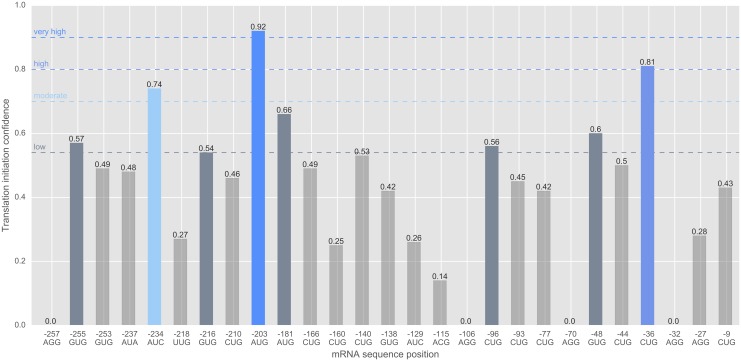
Alternative start codons of human gene *GIMAP5*. Predicted start sites were subdivided into four confidence groups and highlighted by different colors and dashed lines: very high (hot/best candidates with *c* ≥ 0.9), high (0.8 ≤ *c* < 0.9), moderate (0.7 ≤ *c* < 0.8) and low (*t* = 0.54 ≤ *c* < 0.7) initiation confidence *c*. For this gene, we found one hot candidate with a very high confidence value of 0.92 of being a true start site (AUG at position -203).

We applied the final model to a gene of interest, GIMAP5 (ENST00000358647), that was not contained in the human ribosome profiling data. GIMAP5 codes for a GTPase binding GTP (Guanosine TriPhosphate) and is involved in the survival of T–cells [36]. The scan of *GIMAP5* resulted in 27 candidate start sites with an in–frame stop codon and a surrounding window of ±99 nts to calculate the k-mer features in. Fig 5 shows the predicted initiation probabilities of the putative start sites.

Out of these 27 candidate start sites, we found eight start codons with a confidence value above *t* = 0.54. Among these starts, we found one hot candidate (AUG at position -203) with a very high confidence value of 0.92 of being a true start site. Moreover, a CUG at position -36 was also predicted with a high confidence value of 0.81. We postulate that these start sites are able to initiate translation in a specific cell type or cellular condition (for instance cellular stress response).

In this manner, the webservice *PreTIS* can be used to visualize all putative start sites and subsequently to predict unknown translational start sites.

#### *In silico* mutation analysis

**Fig 6:**
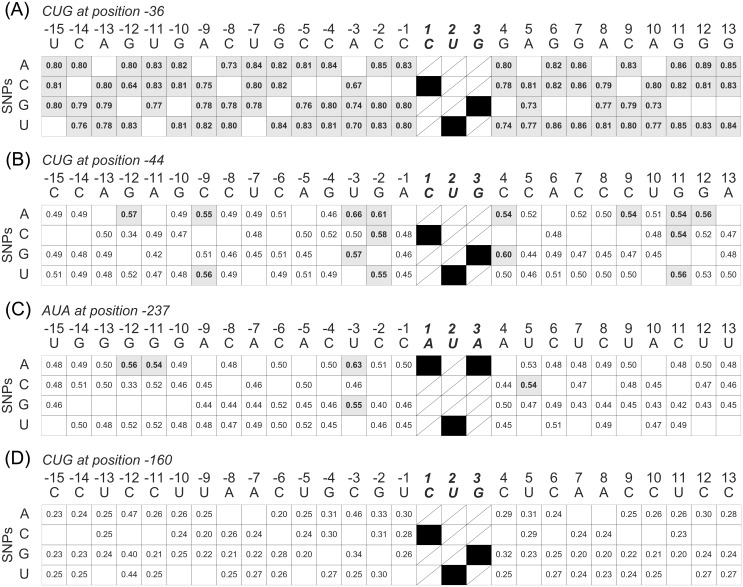
SNP analysis of gene GIMAP5. Mutation matrix showing the impact of the flanking sequence context of four putative start sites of gene *GIMAP5* on the predicted initiation confidence. In each case, only one nucleotide is mutated with respect to the reference sequence (top line). Grey means that the start was predicted as true translational start (predicted initiation confidence is greater than 0.54) whereas white means that the start was classified as false start. Mutations at the start sites itself were not considered. The numbers reflect the predicted initiation confidence values. A: CUG at position -36. B: CUG at position -44. C: AUA at position -237. D: CUG at position -160.

As an outlook where this methodology could be helpful as well, we investigated the effect of fictitious SNPs on the translational initiation confidence around all start sites of gene *GIMAP5*. We used the same surrounding window of -15 to +10 that was used to calculate the PWMs. Fig 6 shows three possible scenarios how *in silico* mutations in the flanking sequence context of a putative start site affect its predicted initiation confidence.

In the first example, the initiation confidence value is, independent of the SNP, always above the threshold. This means that the start site is always predicted as true start site since the overall advantageous properties are not changed severely by a single SNP that is inserted (see Fig 6A).

In the second case, the predicted initiation confidence value changes dependent on the SNP that is artificially inserted into the flanking sequence (see Fig 6B and 6C). Take for instance, CUG at position -44 (Fig 6B): a C\G at position +4 increases the initiation confidence from 0.53 (see Fig 6) to 0.60. The same holds for a U\A SNP and U\G SNP at position -3 that increase the predicted initiation confidence to 0.66 and 0.57, respectively. For the AUA start site at position -237 (Fig 6C), an U\A SNP and a U\G SNP at position -3 increased the initiation confidence from 0.48 to 0.63 and 0.55, respectively. Positions -3 and +4 were mentioned beforehand to be crucial for translation initiation [24, 25]. Moreover, SNPs at position -12, also found to be significant by the k–mer search (Table 3), seem to have an important influence on the translation initiation. A G\C SNP entails a dramatic drop of the initiation confidence value to 0.33 (Fig 6C) since far less true starts contain Cs at position -12 (0.13) compared to false starts (0.3), see Table 3.

Finally, it may also happen that the initiation confidence is always below the given threshold, independent of the SNP that is inserted (Fig 6D). This is based on the overall disadvantageous properties of a start site such that a single mutation cannot “boost” the overall disability of this start to initiation translation. The results of the *in silico* mutation analysis of all other *GIMAP5* start sites can be found in S6 Fig.

**Fig 7:**
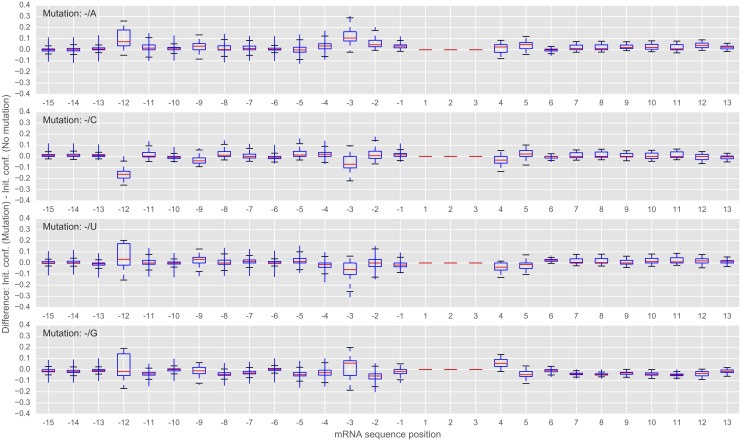
*In silico* mutation analysis considering all 3,566 genes of the HEK293 dataset. The flanking sequences of all possible start sites in the HEK293 dataset were mutated. Shown is the difference in the predicted initiation confidence (*IC*_*difference*_ = *IC*_*mutation*_ − *IC*_*wildtype*_). Positions -3 and -12 are prevalent and seem to have the largest influence on the prediction. Positions at the start site were not mutated.

To investigate the influence on the predicted initiation confidence (IC) on a more general scale, we calculated the difference in the initiation confidence of a mutation (A, C, U and G) compared to the wildtype sequence (*IC*_*difference*_ = *IC*_*mutation*_ − *IC*_*wildtype*_) for all start sites in the 3,566 genes of the HEK293 dataset. The results are shown in Fig 7. For example, if adenine or guanine are inserted at position -3, the initiation confidence value increases, with median values of 0.11 and 0.06, respectively (see Fig 7). As mentioned, position -12 seems to play an important role in translational initiation. By comparing all start sites and possible mutations, a cytosine at this position lowers the initiation confidence by 0.16 on average.

Moreover, it was experimentally shown that positions +5 and +6 are important for efficient translation initiation, especially in non-AUG initiation [37]. More precisely, it was shown that the second codon (i.e. positions 4, 5 and 6) being G**AU** or G**CU** enabled an efficient translation initiation while G**UA** ablated initiation. Thus, an AU or CU seem to be important at position +5/+6 while UA is disadvantageous for translation initiation. This experimental finding can also be observed in Fig 7: A and C at position +5 increase and U at position +5 decreases the confidence value, while on the other hand at position +6, a U increases the confidence value.

## Discussion

We were able to identify highly significant features belonging to three different feature classes (biologically-motivated features, PWM as well as k–mer features) that distinguish between true and false translation initiation sites. A simple linear regression model based on significant and uncorrelated features enabled us to reliably differentiate between true and false start sites. While a k–mer search enabled an unbiased scan, the biologically–motivated features reflect experimental observations regarding translation initiation. The PWM accounts for the flanking sequence context that is crucial to initiate translation. Also, it reflects the role of the start codon itself since it was shown that some codons (e.g. AUG and CUG) are used more often by the ribosome to initiate translation in mammals [3, 4].

Problematic was the inhomogeneous data set that most likely contains some FPs and misses some TPs. Reasons for this may be experimental drawbacks, the processing steps of the raw data, or the cell line that was used (some start sites may only be used in specific cell lines). In general, several experimental steps have an influence on and can alter the data output: cell harvesting, nuclease treatment and library generation [9]. The key idea of ribosome profiling is the inhibition of translation. This may introduce certain biases into the data. For example, if inhibition is slow, ribosomes can artificially accumulate at specific positions [9]. Moreover, RNA fragments (e.g. non-coding RNAs) can distort the translation readouts. Especially in sequence analysis, the mapping of the sequence reads from similar regions of different transcript variants is challenging. This is further complicated by the short length (about 30 nucleotides) of ribosome footprints [9]. Moreover, it is currently not possible to apply ribosome profiling to single cells, in contrast to mRNA-seq for instance [9].

Without doubt, the ribosome profiling technique is a huge innovation to understand translational initiation. However, it appears that the start codon selection based on the experimental outcome is challenging. For example, a GUG start in gene *RPLP1* at position -107 determined by Lee *et al.* (2012) has the following flanking sequence context: GCC GCC AAG GUG CUC [3]. In the light of the findings of Kozak [25, 38, 39], one may speculate whether the upstream AAG codon would be the more appropriate start codon. Nevertheless, the deep analyses of the different datasets presented here was able to point out crucial sequence features for which a solid experimental evidence exists (for example Kozak context) that significantly differed between the considered true and false start sites. This verifies and draws confidence that the overall ribosome profiling dataset(s) are suitable for the prediction of translational start sites.

Although, we used ribosome profiling applied to a specific cell line (HEK293) for training and testing, we propose that the predicted start sites have the potential to initiate translation in other cell types as well since the features used are only based on sequence properties. As a rather extreme example, we showed that the classifier trained on human HEK293 cells works reasonably well, albeit with lower accuracy, on mouse ES cells. Interestingly, we observed that the codon distribution of predicted start sites in the test set was similar to that of the experimentally observed start sites. This provides confidence in the quality of our prediction approach. Moreover, applying regression instead of classification enabled us to provide an initiation confidence value ranging from 0.0 to 1.0 rather than a strict decision between true and false start site. Subdividing start sites into different confidence classes *c* (very high: *c* ≥ 0.9, high: 0.8 ≤ *c* < 0.9, moderate: 0.7 ≤ *c* < 0.8 and low: *t* = 0.54 ≤ *c* < 0.7) helps to identify *hot* candidate start sites with very high initiation confidence values. The analysis of SNPs in the start site flanking sequence context showed that mutations can have a large impact on the initiation confidence. This not only holds true in our prediction approach but also in the context of *in vivo* translation. Kozak found that individual mRNA positions (-3, +4) are crucial for initiation [24, 25].

### Webservice *PreTIS*

*PreTIS* is a webservice to predict the initiation confidence of all reading–frame independent start sites (AUG and all near-cognate codons) located in the 5’ UTR of human mRNA sequences. Thereby, the best human prediction model described here is used as underlying regression model. The webservice application *PreTIS* requires the mRNA sequence and is accessible at http://service.bioinformatik.uni-saarland.de/pretis.

Based on the given human mRNA sequence, all possible AUG and near-cognate start sites, with a surrounding window of at least ±99 nts (needed to calculate k-mers) and an in–frame downstream stop codon, are located in the 5’ UTR. *PreTIS* then calculates the required sequence features (see Table 3) for all detected start sites and subsequently predicts the initiation confidence. Based on the predicted initiation confidence value and the given prediction threshold of *t* = 0.54, a start site is categorized into different initiation confidence classes. For start sites with confidence values *c* greater than the given threshold *t*, the four confidence groups were defined as follows: very high (hot/best candidates with *c* ≥ 0.9), high (0.8 ≤ *c* < 0.9), moderate (0.7 ≤ *c* < 0.8) and low (*t* ≤ *c* < 0.7) confidence, respectively. Especially start sites with very high confidence values can be considered as *hot* candidates for translational initiation.

The predicted initiation confidence for each start site is visualized by barplots with the x-axis displaying the mRNA position (compare to Fig 5). This enables a comprehensive comparison of, for example, different flanking sequence contexts. Features calculated for each start site can also be downloaded as.csv file for further analyses. For the calculation of some features, an orthologous mouse sequence is required. This is automatically implemented by the embedded *blastn* [22] search. The mRNA sequence found by *blastn* can be inspected afterwards and replaced, if desired. Furthermore, each job is given a *Session–* and a *Job–ID*, which enables unambiguous accession to the prediction results.

Thus, *PreTIS* is an intuitive tool that solely requires the human mRNA sequence as input. It gives access to various calculated sequence-encoded and experimentally shown important sequence properties for translational initiation. In addition, an initiation confidence value for each start site is calculated using an established regression model that is based on recent experimental data. AUG as well as alternative start codons—in and out of the main reading frame—are considered.

## Supporting Information

S1 Fig Relative frequency of different start codons. Distribution of different true start codons in human HEK293 cells (4,482 true starts) and in mouse ES cells (3,009 true starts).
(TIFF)

S2 Fig Kozak sequence context. The flanking sequence context of the 4,482 true starts (left columns) and the 49,520 false starts (right columns) with respect to positions -3 and +4, which were shown to be crucial for translation initiation. The definitions of different “Kozak types” are described in the methods section.
(TIFF)

S3 Fig Linear regression model. Linear regression coefficients of the best human linear regression model trained on HEK293 data.
(TIFF)

S4 Fig Different prediction thresholds to classify start sites of the mouse ES dataset. The solid line corresponds to the threshold of *t* = 0.54 whereas the dashed line displays results for *t* = 0.52. A: Unbalanced dataset. B: Balanced dataset.
(TIFF)

S5 Fig Different prediction thresholds to classify start sites of the HEK293–AUG dataset. The solid line corresponds to the threshold of *t* = 0.54 whereas the dashed line displays results for *t* = 0.65 and *t* = 0.64, respectively. A: Unbalanced dataset. B: Balanced dataset.
(TIFF)

S6 Fig GIMAP5 *in silico* mutation analysis results of all start sites. Mutation matrices showing the impact of the flanking sequence context of all putative start sites of gene *GIMAP5* on the predicted initiation confidence. In each case, only one nucleotide is mutated with respect to the reference sequence (top line). Grey means that the start was predicted as true translational start (predicted initiation confidence is greater than 0.54) whereas white means that the start was classified as false start. Mutations at the start sites itself were not considered. The numbers reflect the predicted initiation confidence.
(PDF)
