# Supplementary figures and images for: PreTIS: A Tool to Predict Non-canonical 5’ UTR Translational Initiation Sites in Human and Mouse
Source: PLoS Comput Biol. 2016 Oct 21;12(10):e1005170. doi: 10.1371/journal.pcbi.1005170 (PMC5074520; doi:10.1371/journal.pcbi.1005170)

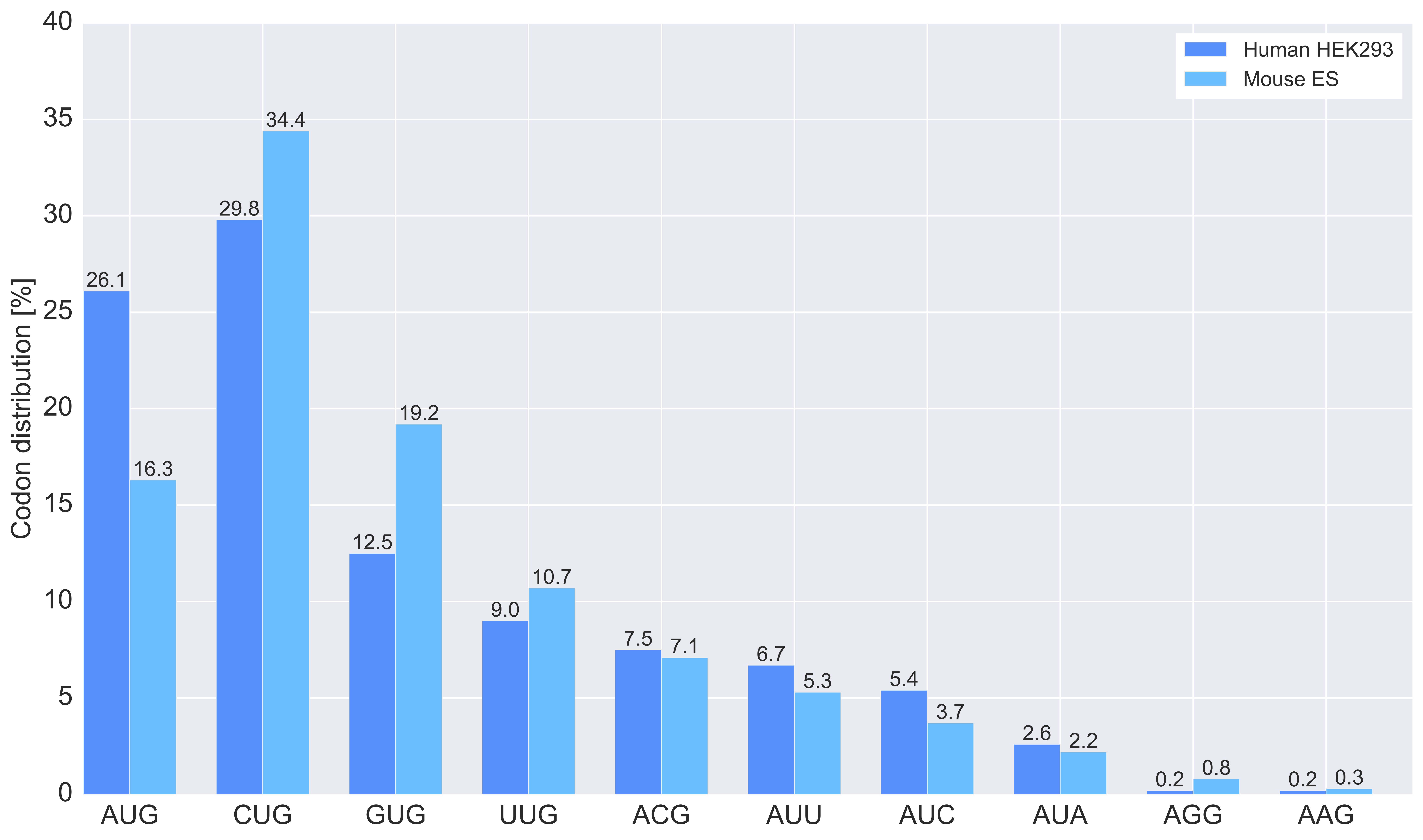

Supplement: S1 Fig — Distribution of different true start codons in human HEK293 cells (4,482 true starts) and in mouse ES cells (3,009 true starts). (TIFF) [file pcbi.1005170.s001.tiff]

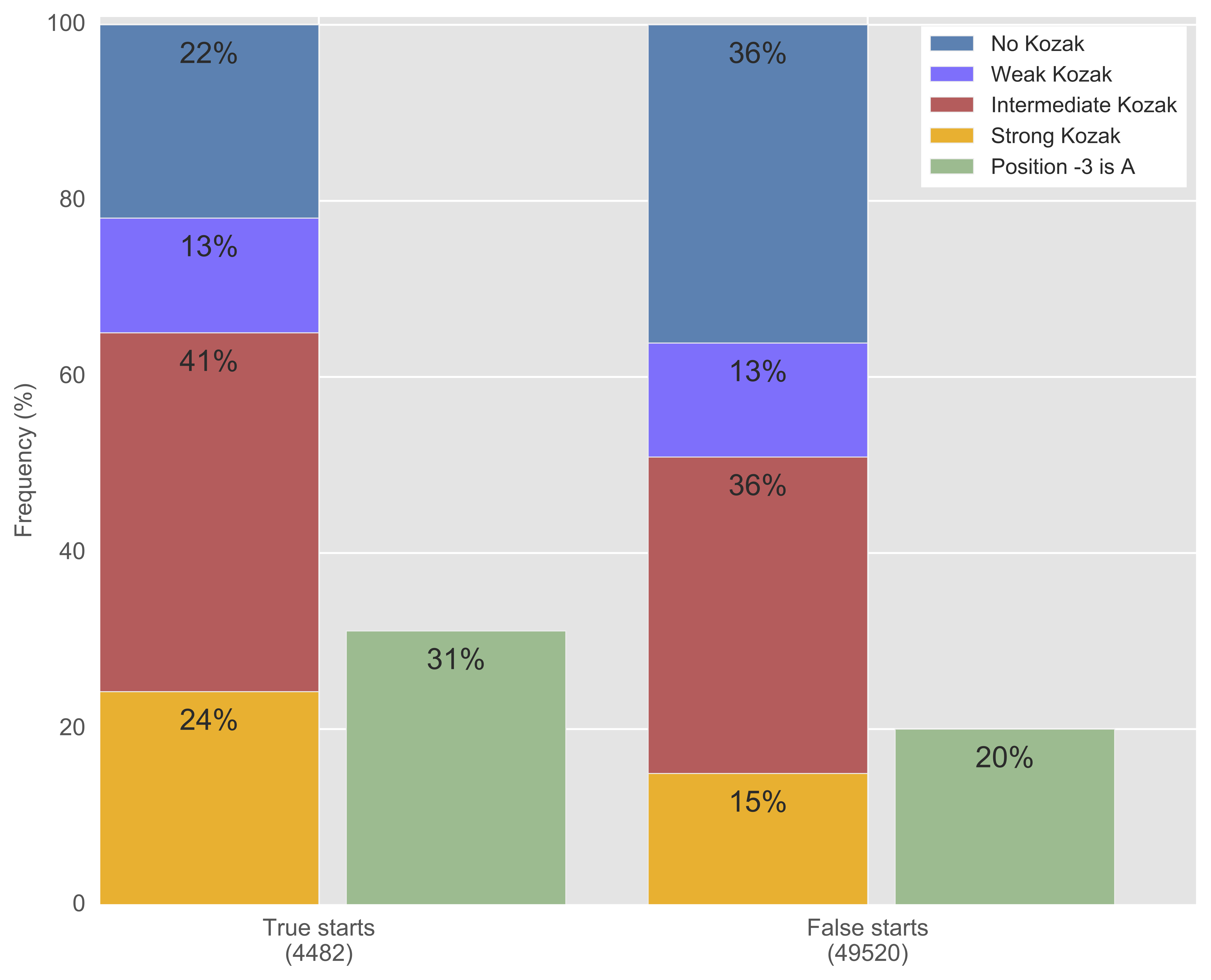

Supplement: S2 Fig — The flanking sequence context of the 4,482 true starts (left columns) and the 49,520 false starts (right columns) with respect to positions -3 and +4, which were shown to be crucial for translation initiation. The definitions of different “Kozak types” are described in the methods section. (TIFF) [file pcbi.1005170.s002.tiff]

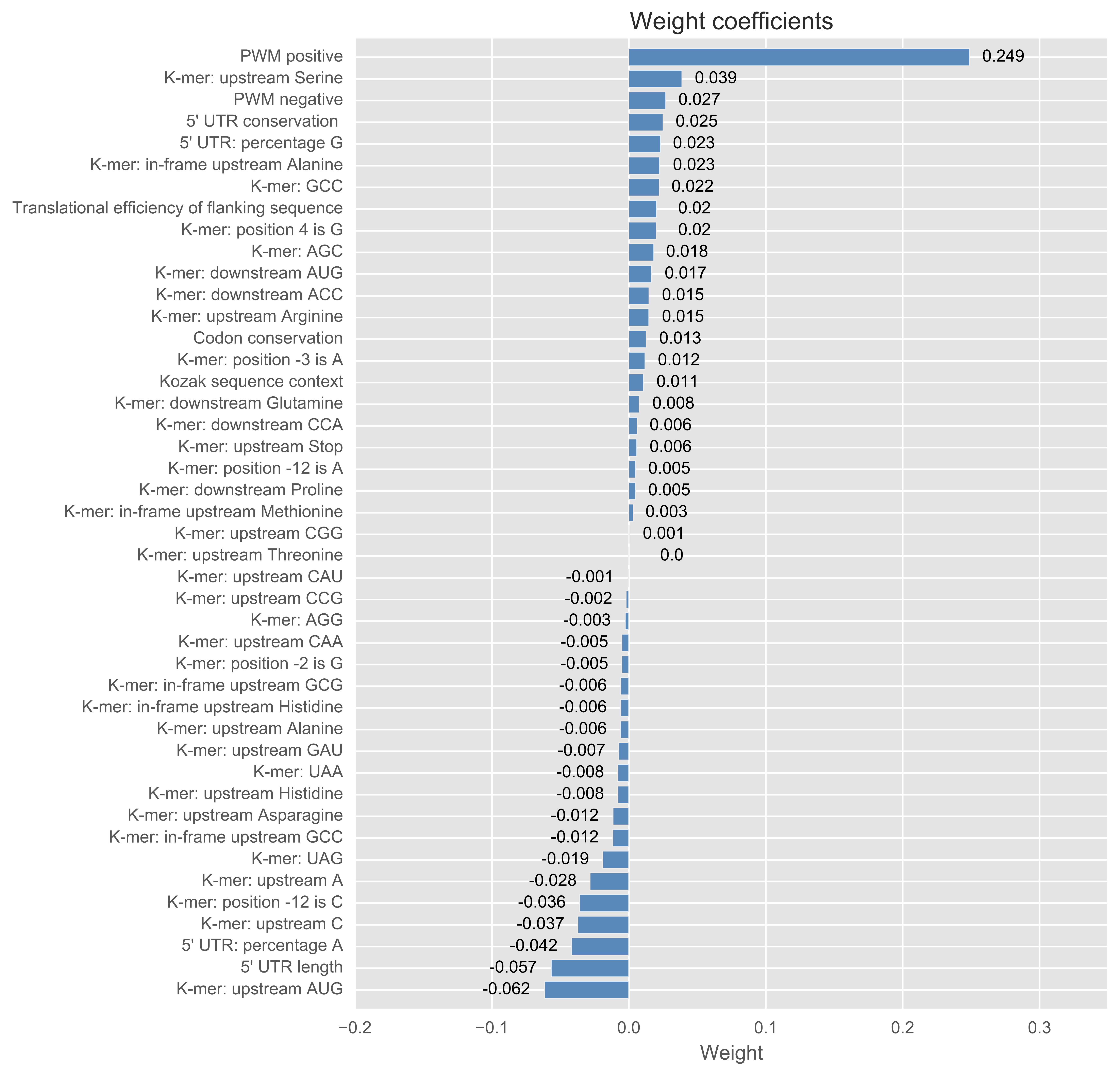

Supplement: S3 Fig — Linear regression coefficients of the best human linear regression model trained on HEK293 data. (TIFF) [file pcbi.1005170.s003.tiff]

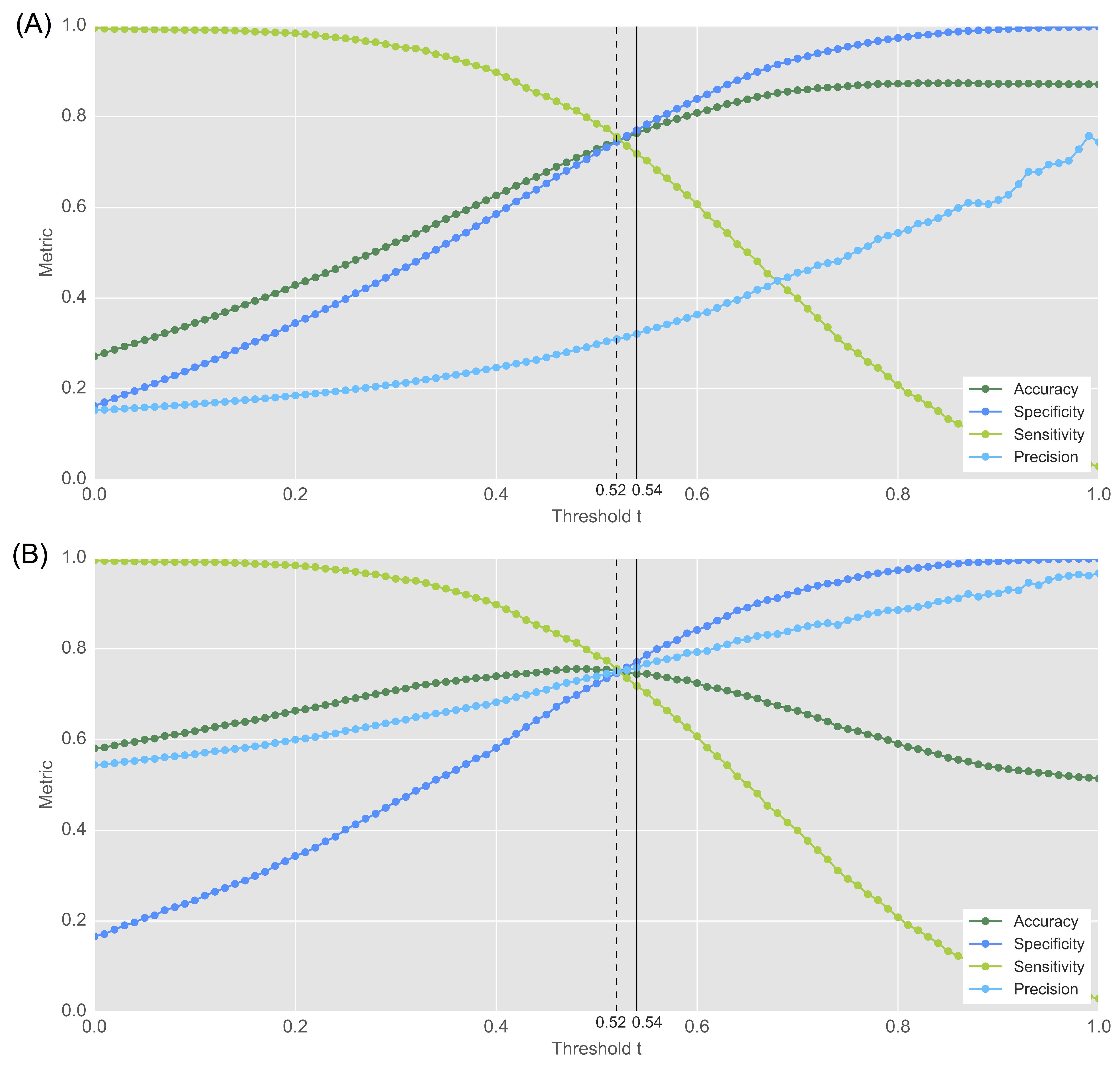

Supplement: S4 Fig — The solid line corresponds to the threshold of t = 0.54 whereas the dashed line displays results for t = 0.52. A: Unbalanced dataset. B: Balanced dataset. (TIFF) [file pcbi.1005170.s004.tiff]

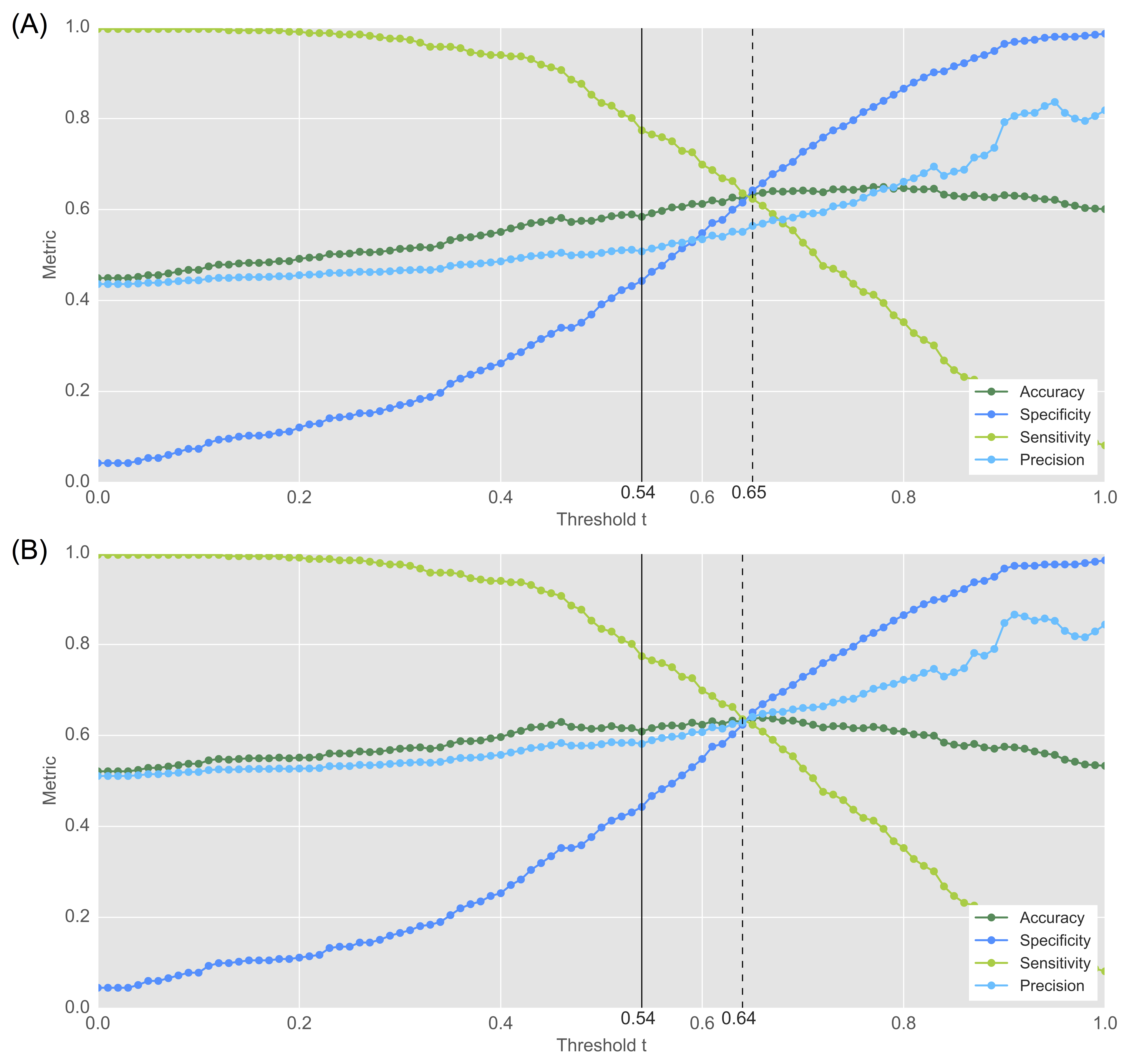

Supplement: S5 Fig — The solid line corresponds to the threshold of t = 0.54 whereas the dashed line displays results for t = 0.65 and t = 0.64, respectively. A: Unbalanced dataset. B: Balanced dataset. (TIFF) [file pcbi.1005170.s005.tiff]
