# Supplementary material for: PreTIS: A Tool to Predict Non-canonical 5’ UTR Translational Initiation Sites in Human and Mouse
Source: PLoS Comput Biol. 2016 Oct 21;12(10):e1005170. doi: 10.1371/journal.pcbi.1005170 (PMC5074520; doi:10.1371/journal.pcbi.1005170)
Supplement: S6 Fig — Mutation matrices showing the impact of the flanking sequence context of all putative start sites of gene GIMAP5 on the predicted initiation confidence. In each case, only one nucleotide is mutated with respect to the reference sequence (top line). Grey means that the start was predicted as true translational start (predicted initiation confidence is greater than 0.54) whereas white means that the start was classified as false start. Mutations at the start sites itself were not considered. The numbers reflect the predicted initiation confidence. (PDF) [file pcbi.1005170.s006.pdf]

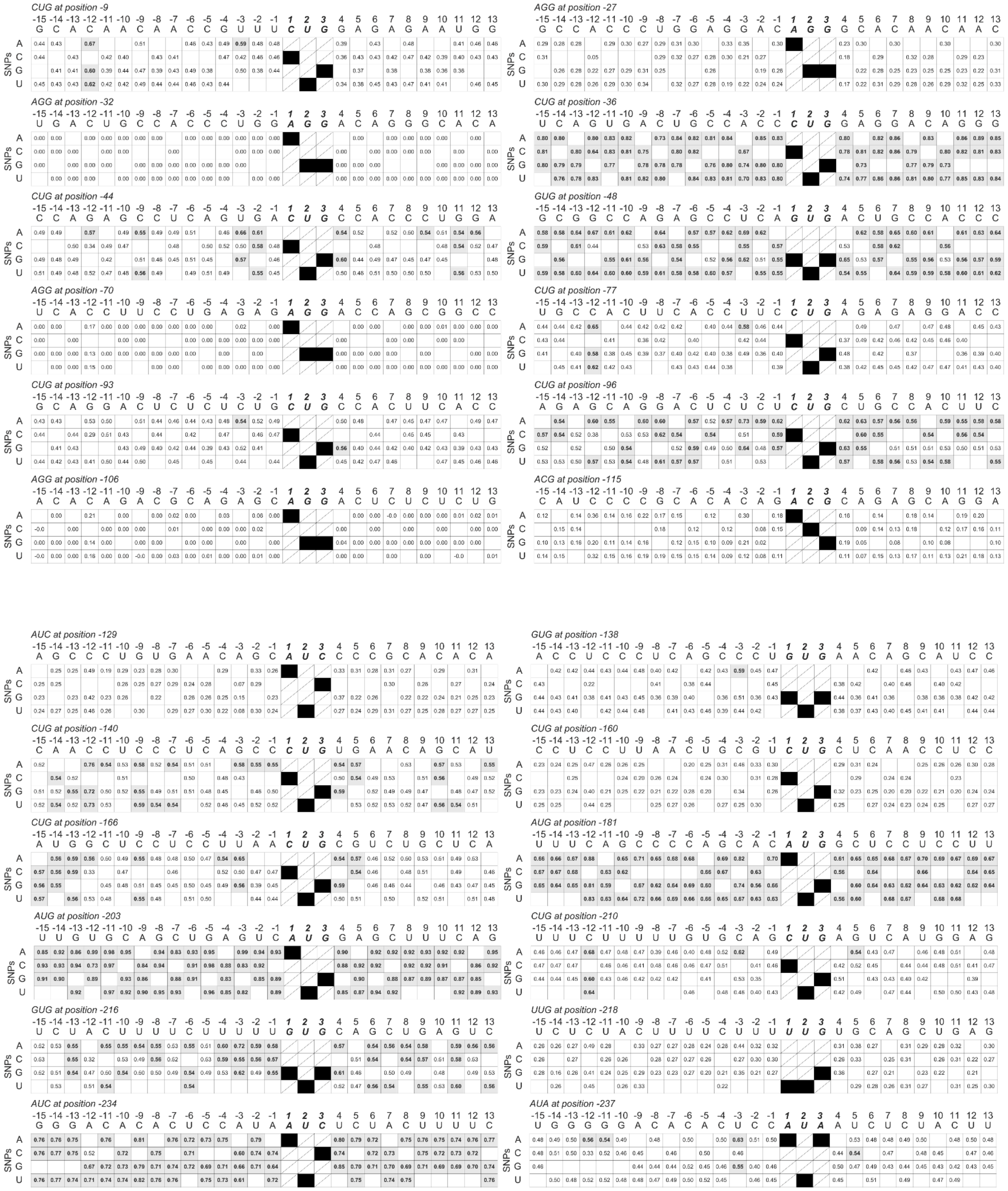
CUG at position -9
AGG at position -27
AGG at position -32
CUG at position -36
CUG at position -44
GUG at position -48
AGG at position -70
CUG at position -77
CUG at position -93
CUG at position -96
AGG at position -106
ACG at position -115
AUC at position -129
GUG at position -138
CUG at position -140
CUG at position -160
CUG at position -166
AUG at position -181
AUG at position -203
CUG at position -210
GUG at position -216
UUG at position -218
AUC at position -234
AUA at position -237
SNPs

GUG at position -253

SNPs	-15	-14	-13	-12	-11	-10	-9	-8	-7	-6	-5	-4	-3	-2	-1	***1***	***2***	***3***	4	5	6	7	8	9	10	11	12	13
	C	U	A	G	U	C	C	G	C	A	G	A	G	G	U	***G***	***U***	***G***	G	G	G	G	A	C	A	C	A	C
A	0.49	0.50		**0.56**	0.49	0.51	**0.58**	0.53	0.52		0.51		**0.58**	**0.57**	0.53				0.43	0.53	0.50	**0.54**		0.51		0.51		0.53
C		0.50	0.50	0.34	0.50			**0.54**		0.48	0.52	0.48	0.39	**0.56**	0.50				0.39	**0.55**	0.49	0.53	0.50		0.49		0.46	
G	0.48	0.49	0.48		0.44	0.49	0.52		0.48	0.49		0.43			0.48								0.45	0.47	0.44	0.46	0.43	0.50
U	0.49		0.48	0.53		0.51	**0.55**	**0.55**	0.50	0.49	0.52	0.43	0.30	**0.54**					0.40	0.48	0.53	**0.54**	0.49	0.50	0.49	0.51	0.48	0.52

AGG at position -257

SNPs	-15	-14	-13	-12	-11	-10	-9	-8	-7	-6	-5	-4	-3	-2	-1	***1***	***2***	***3***	4	5	6	7	8	9	10	11	12	13
	U	G	U	C	C	U	A	G	U	C	C	G	C	A	G	***A***	***G***	***G***	U	G	U	G	G	G	G	G	A	C
A	0.00	0.00	0.00	0.00	0.00	0.00		0.00	0.00	0.00	0.00	0.00	0.00		0.00				0.00	0.00	0.00	0.00	0.00	0.00	0.00	0.00		0.00
C	0.00	0.00	0.00			0.00	0.00	0.00	0.00			0.00		0.00	0.00				0.00	0.00	0.00	0.00	0.00	0.00	0.00	0.00	0.00	
G	0.00		0.00	0.00	0.00	0.00	0.00		0.00	0.00	0.00		0.00	0.00					0.00		0.00						0.00	0.00
U		0.00		0.00	0.00		0.00	0.00		0.00	0.00	0.00	0.00	0.00	0.00					0.00		0.00	0.00	0.00	0.00	0.00	0.00	0.00

GUG at position -255

SNPs	-15	-14	-13	-12	-11	-10	-9	-8	-7	-6	-5	-4	-3	-2	-1	***1***	***2***	***3***	4	5	6	7	8	9	10	11	12	13
	U	C	C	U	A	G	U	C	C	G	C	A	G	A	G	***G***	***U***	***G***	U	G	G	G	G	G	A	C	A	C
A	**0.56**	**0.56**	**0.57**	**0.62**		**0.58**	**0.56**	**0.56**	**0.60**	**0.57**	**0.57**		**0.64**		**0.61**				**0.62**	**0.64**	**0.57**	**0.61**	**0.62**	**0.61**		**0.58**		**0.60**
C	**0.57**			0.39	**0.56**	**0.56**	0.51			**0.56**		**0.54**	0.50	**0.55**	**0.60**				**0.58**	**0.61**	**0.57**	**0.60**	**0.61**	**0.60**	**0.56**		**0.54**	
G	**0.55**	**0.54**	**0.56**	**0.55**	0.51		0.52	0.51	**0.55**		0.52	0.52		0.48					**0.67**						0.52	0.53	0.51	**0.57**
U		**0.57**	**0.56**		**0.55**	**0.57**		**0.57**	**0.58**	**0.59**	**0.56**	0.53	0.50	**0.54**	**0.58**					**0.62**	**0.61**	**0.62**	**0.62**	**0.60**	**0.56**	**0.58**	**0.55**	**0.59**
